# *PRKRAP1* and Other Pseudogenes in Movement Disorders: The Troublemakers in Genetic Analyses Are More Than Genomic Fossils

**DOI:** 10.1002/mdc3.13499

**Published:** 2022-06-23

**Authors:** Francesca Magrinelli, Katja Lohmann

**Affiliations:** ^1^ Department of Clinical and Movement Neurosciences, UCL Queen Square Institute of Neurology University College London London; ^2^ Institute of Neurogenetics University of Lübeck Lübeck Germany

**Keywords:** *GBA*, *SMN2*, *PRKRA*, next‐generation sequencing, pseudogene.

The term pseudogene (“pseudo‐” from Ancient Greek pseudés, “fake, mimic”) was coined by Jacq et al^1^ in 1977 to indicate a nearly identical reproduction of the 5S ribosomal RNA gene paired with the gene itself in a unit tandemly repeated in *Xenopus laevis*. Thenceforth, similar gene multiplications were recognized for actin genes in *Dictyostelium discoideum*,^2^ globin genes in mammals,^3^ and small nuclear RNA genes in humans.^4^

By the original definition, a pseudogene is a DNA sequence that resembles a gene, but has accumulated (disrupting) variants over the course of evolution. Therefore, pseudogenes, which always derive from functional genes,^5^ are unable to produce functional proteins due, for instance, to altered open reading frames (ORF) with frameshift or nonsense mutations. Recently, next‐generation sequencing (NGS) and advanced bioinformatics algorithms have enabled the interrogation of DNA sequences at an unprecedented pace, with pseudogenes being systematically detected throughout the genome of most eukaryotic organisms.^6^ To date, the reference annotation project GENCODE (v39) lists ~15,000 pseudogenes in the human genome (https://www.gencodegenes.org/human/stats.html).^7^ Although several genes in the human genome have one pseudogene, a few gene families contain an incredibly high number of pseudogenes, including ribosomal proteins (~80 genes, >2000 pseudogenes)^8^ and olfactory receptor genes.^9^

Pseudogenes in eukaryotic genomes are detected by computational pipelines and manual annotation. Bioinformatics tools for pseudogene prediction have been developed and publicly released.^10^, ^11^ Pseudogenes are primarily identified by comparing their sequence with that of their parental genes, with possible lack of introns and disruptions to the ORF relative to the parental gene being the primary features used to identify pseudogenes. Pseudogenes can be divided into two major groups, that is, unprocessed pseudogenes and processed pseudogenes, with the latter representing the majority of pseudogenes in humans (~70%, https://www.gencodegenes.org/human/stats.html). In brief, unprocessed pseudogenes arise from duplication of genomic DNA sequences and lie on the same chromosome as their parental gene. Processed pseudogenes derive from messenger RNA (mRNA) retrotransposition and are usually located on a different chromosome than the parental gene (Fig. 1A).

**FIG. 1:**
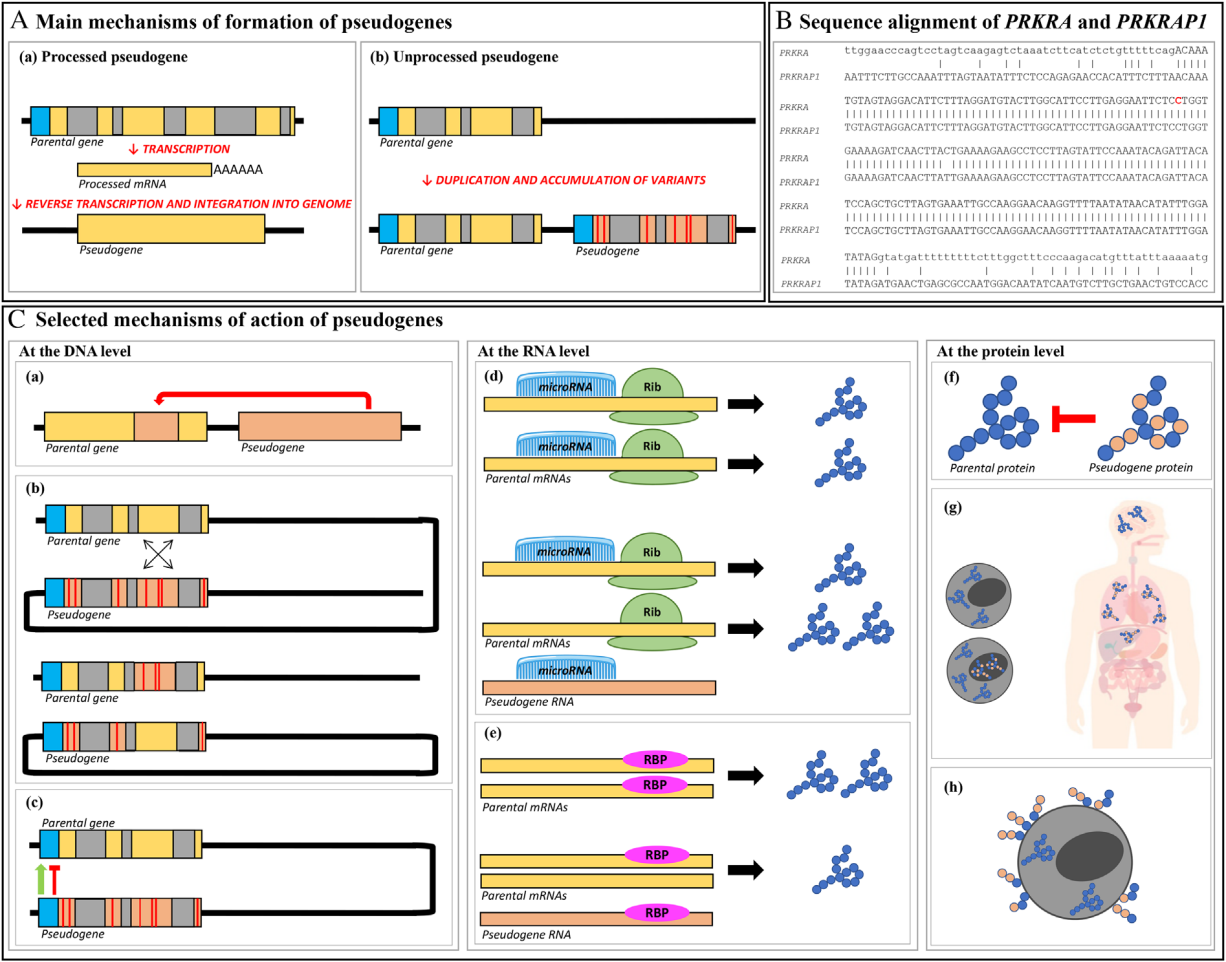
Types and functions of pseudogenes. (**A**) Main mechanisms of formation of pseudogenes. (**A‐**a) Processed pseudogenes derive from reverse transcription of processed messenger RNA (mRNA) from parental genes followed by reintegration of DNA into the genome, therefore, lacking regulatory regions and introns. (**A**‐b) Unprocessed pseudogenes arise from gene duplication of the parental gene and subsequent accumulation of variants and therefore, do contain introns and may contain regulatory regions depending on the respective break points. Blue boxes represent regulatory regions; yellow and orange boxes are exons with and without coding potential, respectively; gray boxes are introns; red lines symbolize mutations. (**B**) Sequence alignment of *PRKRA* and its pseudogene *PRKRAP1*. The sequence of exon 7 (in upper case letters) of *PRKRA* and the adjacent intronic sequence (in lower case letters) is shown in the upper line and aligned to the sequence of *PRKRAP1* (lower line). There is a high homology in the coding part as indicated by dashes but not for the intronic regions. The c.665C>T mutation is highlighted in red. (**C**) Selected functions of pseudogenes at the DNA, RNA, and protein level. (**C**‐a) Gene conversion consists of a monodirectional transfer of DNA from a (processed) pseudogene to its parental gene leading to changes in the sequence of the latter. (**C**‐b) Homologous recombination between the pseudogene and its parental gene leads to accumulation of variants also in the parental gene. This is what is often seen in the *GBA* gene. (**C**‐c) Regulatory sequences of the pseudogene can positively or negatively interfere with the normal transcription of the parental gene. (**C**‐d) Pseudogene RNAs compete with their parental mRNAs for shared microRNAs, therefore, changing the expression of the parental gene (here shown as enhancement). (**C**‐e) Pseudogene RNAs compete with their parental mRNAs for shared stabilizing RNA‐binding proteins (RBPs), therefore, inhibiting the expression of the parental gene. (**C**‐f) Pseudogene‐derived proteins with partial or altered function affect the activity of their parental proteins. (C‐g) Pseudogene‐derived proteins function as the parental proteins, but in different tissues, cellular compartments, or pathophysiological conditions. (C‐h) Antigenic peptides derived from short pseudogene's open reading frames are exposed on the cell surface. Blue boxes represent regulatory regions; yellow and orange boxes are exons with and without coding potential, respectively; gray boxes are introns; red lines are mutations; green structures represent ribosomes; blue chains symbolize functional proteins, whereas blue and orange chains symbolize pseudogenic proteins.

The high sequence similarity of parental genes, in which mutations can lead to human disease and the non‐coding pseudogene(s), can pose a challenge to genetic testing, especially when using short‐read NGS‐based assessments. In this issue of *Movement Disorder Clinical Practice*, Ribeiro et al^12^ report how they mastered this challenge when they established a correct molecular diagnosis despite targeting a pseudogene, which made trouble by overlaying the genetic cause. Specifically, they describe a case of now genetically proven DYT‐*PRKRA* (formerly DYT16) with the typical phenotype consisting of childhood‐onset, generalized dystonia.^12^, ^13^ Biallelic mutations in *PRKRA* have undoubtedly been linked to a recessively inherited form of early‐onset generalized dystonia.^14^ They acknowledge that the delayed molecular diagnosis in their patient on NGS‐based gene panel analysis was because of incorrect alignment of wild‐type NGS reads from the pseudogene *PRKRAP1* to the parental *PRKRA* gene.^12^ This led to the interpretation of the pathogenic *PRKRA* variant NM_003690:c.665C>T; p.(Pro222Leu) being present in the heterozygous state only until Sanger sequencing unraveled the variant was actually present homozygously.^12^

Second‐generation, short‐read NGS that is used for panel and exome sequencing analyses is particularly prone to alignment mistakes in homologous chromosomal regions. This is based on the nature of short‐read NGS where fragments of only 150 to 300 base pairs (bp) are generated and can show a perfect match between the parental gene and the pseudogene. This is illustrated in Figure 1B for exon 7 of *PRKRA* that shows 99.4% sequence identity (174/175 identical bp) to the *PRKRAP1* pseudogene including 55 nucleotides upstream and 119 nucleotides downstream of the c.665C>T mutation. In contrast, the intronic sequences of *PRKRA* do not show sequence similarity to its pseudogene (Fig. 1B) because *PRKRAP1* as a processed pseudogene does not contain intronic sequences. For Sanger sequencing, the primers are usually located in the intronic regions and designed in a way to be specific for the parental gene. However, only short reads are possible for second‐generation sequencing because of the so‐called sequencing‐by‐synthesis technology.

In a first step, the NGS workflow includes library preparation by fragmenting genomic DNA in short size‐uniform pieces of double‐stranded DNA, followed by ligating technology‐specific adapters to both fragment ends, and subsequent amplification and sequencing of these DNA fragments to generate millions of “reads.” After these steps, reference‐based bioinformatics pipelines include a mandatory “alignment” stage before downstream algorithms can be run. Bioinformatics tools, such as the industry standard “Burrows‐Wheeler Aligner” (BWA),^15^ can execute alignment and map (ie, report the positional genomic coordinates of) NGS reads onto the indexed reference genome. Most alignment algorithms nowadays score each seed alignment based on matches, mismatches, or gaps between each read and its assigned reference genomic position, so that the highest score corresponds to the primary alignment for that specific read. Primary alignment can, however, be assigned erroneously when, for instance, a correct alignment of reads containing common polymorphisms is scored lower than an incorrect one characterized by fewer mismatches. For this reason, alignment of the short reads from highly homologous genomic loci, such as genes and their corresponding pseudogenes, are particularly at risk to be misaligned to one or the other as was the case for *PRKRA* and *PRKRAP1*.^12^

Pseudogenes were once regarded as “genomic fossils,” that is, functionless fragments of protein‐coding genes being incorporated into the genome.^16^, ^17^ Although most pseudogenes in the human genome have not been characterized for biological functions, growing evidence suggest that many of them have important biological and genetic roles that are sometimes useful and sometimes harmful. In some cases, the duplication does not result in a complete loss‐of‐function of the duplicated gene, but rather serves as a backup copy with at least some compensatory function. This is for instance the case for *SMN2*, which originates from an inverse duplication of the *SMN1* (survival motor neuron protein) locus. Biallelic variants in *SMN1* cause spinal muscular atrophy (SMA). *SMN2* differs from *SMN1* by five nucleotides, none of which changes the encoded protein sequence, but one affects splicing, therefore, resulting in only ~10% of *SMN2* transcripts including exon 7 and hence, full‐length SMN protein.^18^ Most importantly, *SMN2* has become the target of bifunctional antisense oligonucleotides preventing exon 7 skipping and ultimately rescuing SMN synthesis in a licensed disease‐modifying treatment for SMA (nusinersen).^19^

Pseudogenes can act on their parental genes and alter their sequence at the DNA level, or their expression and functionality on the mRNA and protein level through several mechanisms (Fig. 1C).

At the DNA level, for instance, the high homology between a pseudogene and its parental gene predisposes to non‐allelic homologous recombination leading to a wide range of structural variants (Fig. 1C). Importantly, such disruption of the functionality of the parental gene can lead to human diseases. In the field of movement disorders, paradigmatic is the case of *GBA* variants, which are recognized as the single largest risk factor for the development of Parkinson's disease. *GBA* encodes the lysosomal enzyme glucocerebrosidase. Detecting *GBA* variants is challenging because of its neighboring, unprocessed pseudogene (*GBAP1*), which has an overall homology of 96% with *GBA*. In particular, the homology rate peaks at 98% in the region from intron 8 to the 3′‐UTR, where five identical segments >200 bp each are recognized. Homologous recombinations between *GBA* and *GBAP1* have led to the generation of well‐established “complex” structural variants in *GBA* (termed as Rec1 to Rec7),^20^ mainly including conversions and fusions. Pathogenic variants, including these recombinations, can cause Gaucher's disease and be a risk factor for Parkinson's disease, therefore, representing a prime example for a pseudogene causing or predisposing to disease in humans.^21^ The high homology of *GBA* and *GBAP1* also poses challenges on the sequence analysis even when using Sanger sequencing of *GBA*.^22^, ^23^

At the mRNA level, pseudogenes are capable to regulate the expression of their parental genes by competitively binding to microRNAs (ie, short non‐coding RNAs that bind to their target RNAs and repress protein production post‐transcriptionally).^24^ Furthermore, they may generate endogenous small interfering RNAs that downregulate the expression of functional genes. (Fig. 1C).

The definition of a pseudogene has now broadened to include any DNA sequence that is similar to a known gene and has lost some of its original functionality.^25^ Therefore, pseudogenes can be translated into proteins, as recently proven for 140 human pseudogenes, and also act at the protein level.^26^ For instance, pseudogene‐derived proteins may have the same activity as the parental proteins, but function in different tissues, cellular compartments, or pathophysiological conditions. Short ORFs within pseudogenes can be translated and generate antigenic peptides that are exposed on the cell surface triggering immune response or altering identity and therefore, recognition of such cells (Fig. 1C).

Apart from *GBA* and *PRKRA*, a potential role of pseudogenes has not (yet) caught our attention in the field of movement disorders, although several well‐established disease‐linked genes also have pseudogenes. For instance, there is a processed pseudogene of the myoclonus‐dystonia‐linked gene *SGCE* showing ~80% sequence similarity to *SGCE* overall and at most ~90% within an exon. Furthermore, the *ACTB* gene, which encodes β‐actin and in which mutations can cause another form of dystonia, has at least six processed pseudogenes with <93% sequence identity overall, not reaching >95% at the exon level. To cause misalignment in NGS‐based sequence analysis, there should be <3 mismatches per 150‐nucleotide read (ie, >98% sequence identity). Therefore, the sequence identity for these pseudogenes should be too less to seriously trouble sequence analyses.^27^

Overall, pseudogenes have emerged as a hot topic because they can challenge genetic testing and trigger mutational events even in biologically functional parental genes. In the genetics of movement disorders, they have mainly been brought into play regarding *GBA*, but their role might actually be underestimated. As illustrated by the DYT‐*PRKRA* example,^12^ misalignment can lead to false‐negative genetic testing results, especially when using high‐throughput short‐read NGS. Geneticists should be aware of this challenge and address it either by using alternative screening methods such as Sanger sequencing for selected regions, or long‐read sequencing, as proposed for *GBA* analysis.^28^ Notably, the discovery that pseudogenes can have biological functions and interfere with variant calling in NGS‐based diagnostics has opened up to their constant revision for possible reclassification (eg, as protein‐coding or modifier genes) and has enhanced interest in their accurate annotation as part of improved bioinformatics analysis.

## Author Roles

(1) Research project: A. Conception, B. Organization, C. Execution; (2) Data Analysis: A. Design, B. Execution, C. Review and Critique; (3) Manuscript Preparation: A. Writing of the First Draft, B. Review and Critique.

F.M.: 1A, 1B, 1C, 3A, 3B

K.L.: 1A, 1B, 1C, 3A, 3B

## Disclosures

**Ethical Compliance Statement:** We confirm that we have read the Journal's position on issues involved in ethical publication and affirm that this work is consistent with those guidelines. The authors confirm that the approval of an institutional review board was not required for this work. We confirm that no consent for patients was requested for the present article.

**Funding Sources and Conflicts of Interest**: The authors declare that there are no funding sources or conflicts of interest relevant to this work.

**Financial Disclosures for the Previous 12 Months**: F.M. is supported by the Michael J. Fox Foundation (MJFF) and Edmond J. Safra Foundation and was supported by the research grant “Fondo Gianesini” in collaboration with UniCredit Foundation and University of Verona, Italy (February 2021–January 2022). K.L. has been receiving funding from the German Research Foundation (FOR 2488), the Damp foundation, the Parkinson's foundation, and is a member of the GP2 consortium (funded through the MJFF).
